# Using PIM-Taiwan, PRISCUS, and Beers criteria to assess potentially inappropriate medication use among older adults with 90-day rehospitalization: a population-based study in Taiwan

**DOI:** 10.3389/fphar.2023.1194537

**Published:** 2023-07-13

**Authors:** Kun-Pin Hsieh, Ru-Yu Huang, Yi-Hsin Yang, Pei-Shan Ho, Kuang-Peng Chen, Chun-Liong Tung, Ya-Lan Chu, Jui-Hsiu Tsai

**Affiliations:** ^1^ School of Pharmacy, College of Pharmacy, Kaohsiung Medical University, Kaohsiung, Taiwan; ^2^ Department of Pharmacy, Kaohsiung Medical University Hospital, Kaohsiung Medical University, Kaohsiung, Taiwan; ^3^ National Institute of Cancer Research, National Health Research Institutes, Tainan, Taiwan; ^4^ Department of Medical Research, Kaohsiung Medical University Hospital, Kaohsiung Medical University, Kaohsiung, Taiwan; ^5^ Department of Oral Hygiene, College of Dental Medicine, Kaohsiung Medical University, Kaohsiung, Taiwan; ^6^ School of Dentistry, College of Dental Medicine, Kaohsiung Medical University, Kaohsiung, Taiwan; ^7^ Department of Psychiatry, Dalin Tzu Chi Hospital, Buddhist Tzu Chi Medical Foundation, Chiayi, Taiwan; ^8^ Department of Pharmacy, Dalin Tzu Chi Hospital, Buddhist Tzu Chi Medical Foundation, Chiayi, Taiwan; ^9^ School of Medicine, Tzu Chi University, Hualien, Taiwan

**Keywords:** potentially inappropriate medication, 90-day rehospitalization, PIM-Taiwan, PRISCUS, Beers criteria, older adults, Taiwan

## Abstract

**Background:** Multimorbidity and polypharmacy increase the risk of hospitalization in older adults receiving potentially inappropriate medication (PIM). The current study compared the ability of PIM-Taiwan, PRISCUS, and Beers criteria to predict 90-day rehospitalization in older patients with and without PIM.

**Methods:** The retrospective cohort study used Taiwan’s Longitudinal Health Insurance Database to retrieve quarterly information about prescribed medication for adults aged ≥65 years hospitalized between 2001 and 2018. We analyzed the association of PIM with 90-day rehospitalization using logistic regression.

**Results:** The study cohort included 206,058 older adults (mean age: 72.5 years). In the analysis, 133,201 (64.6%), 97,790 (47.5%), and 147,450 (71.6%), were identified as having PIM exposure in PIM-Taiwan, PRICUS, and Beers criteria, respectively. PIM-Taiwan criteria found exposure to PIM affecting the cardiovascular (adjusted OR [aOR] 1.37, 95% confidence interval [CI] = 1.32–1.41), gastrointestinal (aOR 1.26, 95% CI = 1.23–1.30), central nervous (aOR 1.11, 95% CI = 1.08–1.14), and respiratory (aOR 1.16, 95% CI = 1.12–1.20) systems significantly increased the risk of 90-day rehospitalization, after adjustment for covariates. In PRISCUS criteria, exposure to PIM affecting the respiratory (aOR 1.48, 95% CI = 1.41–1.56), central nervous (aOR 1.12, 95% CI = 1.09–1.15), and cardiovascular (aOR 1.20, 95% CI = 1.16–1.24) systems significantly increased the risk. In Beers criteria, exposure to PIM affecting the cardiovascular (aOR 1.37, 95% CI = 1.32–1.41), gastrointestinal (aOR 1.38, 95% CI = 1.35–1.42), central nervous (aOR 1.18, 95% CI = 1.15–1.21), endocrine (aOR 1.10, 95% CI = 1.06–1.15), and respiratory (aOR 1.09, 95% CI = 1.04–1.13) systems significantly increased the risk. Patients with 90-day rehospitalization had higher rates of the potentially harmful drug-drug interaction (DDI) pairs of serotonin syndrome (n = 19; 48.8%), QT prolongation (n = 4; 30.8%), extrapyramidal symptoms (EPS) (n = 102; 24.5%), and hypokalemia (n = 275; 20.1%).

**Conclusion:** Beers criteria was more efficient in predicting 90-day rehospitalization among older adults experiencing PIM in Taiwan than either PIM-Taiwan or PRISCUS. The risk of 90-day rehospitalization was associated with the potentially harmful DDI classes of serotonin syndrome, QT prolongation, EPS, and hypokalemia.

## Introduction

World population aging is growing rapidly in both developed and developing countries. In Taiwan, the proportion of the older adults increased from 7% in 1993 (an aging country) to 14% in 2018 (an aged country), and is predicted to reach 20% in 2025 (a super-aged country) (National Development Council, 2023). This accelerated aging brings a heavy burden of healthcare for older adults in Taiwan (Chen, 2010). In addition, multimorbidity and polypharmacy among older adults are associated with high rates of unplanned hospitalization, particularly for adverse drug reactions (ADRs) due to age-related alterations in pharmacokinetics, pharmacodynamics, and drug-disease interactions (Leendertse et al., 2008; Olivier et al., 2009; Hamilton et al., 2011; Dalleur et al., 2012). Therefore, adequate drug treatment is paramount to forestall preventable hospitalizations in older adults, particularly the frail and those with multiple morbidities.

Potentially inappropriate medication (PIM) use is not a rare event in older adults, and is associated in this population with ADRs, hospitalization, functional decline, and even death (Lau et al., 2005; Passarelli et al., 2005; Lin et al., 2008; Bradley et al., 2012). For this reason, many countries have built their own criteria systems for PIM, because national pharmacotherapeutic guidelines vary in terms of the specific drugs approved (Chang et al., 2014). The different criteria may lead to wide variations in reported PIM prevalence and their associated health-related outcomes (Chang et al., 2014). Of all PIM criteria the Beers criteria, first established in 1991, is the most widely used to detect PIM in older adults as an indicator of geriatric healthcare quality (Wenger and Shekelle, 2001; Pugh et al., 2006; Chang et al., 2011; Dimitrow et al., 2011; Corsonello et al., 2012). In Taiwan, the PIM-Taiwan, introduced in 2012, outlines the relevant and country-specific PIM criteria (Chang et al., 2012; Chang et al., 2019). The PIM-Taiwan criteria have proven their applicability in several cross-sectional studies among older Taiwanese adults (Chang et al., 2014; Chang et al., 2015; Chang et al., 2018). In comparison with the Beers criteria and PRISCUS criteria, PIM-Taiwan can detect a similar number of PIMs across different populations in Taiwan. PIM users had higher health resource utilization and higher costs of medications than non-PIM users (Chang et al., 2014; Chang et al., 2015). Therefore, PIM-Taiwan criteria can be an important tool to reduce PIM-related adverse events in older Taiwanese adults. In Europe, the PRISCUS criteria, based on expert knowledge developed in Germany, helps physicians make individualized therapeutic decisions for their patients (Holt et al., 2010; Fromm et al., 2013; Schubert et al., 2013). Most studies related to PIM criteria have focused on the prevalence of PIM in limited small-to-medium-sized samples, such as inpatient populations or nursing home residents (Reich et al., 2014; Garcia et al., 2015). However, few studies have assessed the association of PIM with health-related outcomes using nationwide samples (Mekonnen et al., 2021).

Based on National Health Insurance (NHI) claims data, the current study was conducted to estimate the prevalence and changes in PIM among older patients before and after a hospitalization as measured by PIM-Taiwan, PRISCUS, and Beers criteria. We sought to investigate the association of PIM via these criteria systems with the risk of 90-day rehospitalization in this population. Furthermore, we also analyzed the risk of hospitalization associated with different classes of potentially harmful drug-drug interactions (DDIs).

## Materials and methods

### Data source and study population

The retrospective cohort study was performed using claim-based data from the 2010 Longitudinal Generation Tracking Database (LGTD 2010), the Catastrophic Illness Registry Dataset (CIRD), the Taiwan Cancer Registry (TCR) database, and Multiple Causes of Death data from 2000 to 2018. Detailed descriptions of the aforementioned 3-dataset sample and study procedures have previously been published and the representativeness of LGTD and TCR has been validated (Chang et al., 2015; Chiang et al., 2015; Hu et al., 2016; Chiang et al., 2017; Center for Biomedical Resources of NHRI, 2023). The LHTG 2010 is a subset of the National Health Insurance Research Database (NHIRD) composed of 2 million randomly-sampled beneficiaries (nearly 10% of the total Taiwanese population) drawn in 2010. The NHIRD was developed and managed by Taiwan’s National Insurance program, which was introduced in 1995 and provides for approximately 99.9% of Taiwanese residents. The LGTD 2010 includes comprehensive claims data for reimbursements for ambulatory, inpatient, emergency, and Chinese medicine visits, and is used to gather information on prescriptions and comorbidities (Center for Biomedical Resources of NHRI; Chiang et al., 2017; Hu et al., 2016). The CIRD contains information about catastrophic illness in patients who suffered from at least one of 30 specific severe medical conditions such as cancer, chronic mental illness (schizophrenia, bipolar I disorder, major depressive disorder), hemodialysis, systemic autoimmune diseases, and stroke (Chang et al., 2015). The TCR provides archives of information on cancer diagnosis and additional Supplementary Material (Chang et al., 2015). The Multiple Causes of Death data provides information on the cause(s) and date of death.

We enrolled patients aged 65 years or older at the first hospitalization from 2001 to 2018 in Taiwan, excluding those with a history of concomitant cancer or catastrophic illness based on the CIRD and TCR records. Patients with at least one hospitalization recorded during the 2001–2018 study period were considered for the first hospitalization. We followed those patients from the first hospitalization until 90 days after discharge or until rehospitalization within this period.

### Measurements

#### Definition of study outcome

The study outcome of 90-day rehospitalization was defined as one of the following events within 90 days after the first discharge: readmittance to the hospital, emergency department visit, hospitalization visa in the emergency department, or death.

#### Potentially inappropriate medication (PIM) exposure

We assessed medication use within 30 days before the first hospitalization (prehospitalization) and within 30 days after the first discharge (post-discharge). Postdischarge was defined as the 30 days following the discharge date, or the date of readmittance within 30 days after discharge, whichever came first. The medications used were classified as PIM or not PIM according to three criteria systems: the updated 2018 PIM-Taiwan criteria (Chang et al., 2018), the updated 2019 Beers criteria (By the American Geriatrics Society Beers Criteria^®^ Update Expert Panel, 2019), and the entire PRISCUS criteria (Holt et al., 2010) (Supplementary Tables S1–S3) (Table 1). Exposure to PIM was considered if the prescribed drug was represented in any of the criteria, and PIM items were also counted. PIM was identified in the database via Anatomical Therapeutic Chemical code and further classified by human body systems, including cardiovascular, endocrine, gastrointestinal, genitourinary, musculoskeletal, central nervous, and respiratory systems; and the additional classifications (not used in all criteria systems) of sex hormones, anti-infective, and blood and blood-forming organs.

**TABLE 1:** Baseline characteristics of the study population at the first hospitalization.

	Total	PIM-Taiwan	Beers	PRISCUS
Variable	n (%)	n (%)	n (%)	n (%)
	206,058	133,201 (64.6)	147,450 (71.6)	97,790 (47.5)
Age				
Mean ± SD	72.5 ± 6.5	72.5 ± 6.4	72.5 ± 6.4	72.7 ± 6.4
Gender				
Male	102,547 (49.8)	64,304 (48.3)	72,954 (49.5)	47,820 (48.9)
Female	103,511 (50.2)	68,897 (51.7)	74,496 (50.5)	49,970 (51.1)
Geographic region				
Taipei district	68,735 (33.4)	42,767 (32.1)	48,550 (32.9)	32,285 (33.0)
North district	28,176 (13.7)	18,034 (13.5)	20,088 (13.6)	13,083 (13.4)
Central district	36,294 (17.6)	24,659 (18.5)	26,994 (18.3)	17,874 (18.3)
South district	33,624 (16.3)	21,956 (16.5)	24,069 (16.3)	15,964 (16.3)
Kaohsiung/Pingtung district	33,186 (16.1)	21,745 (16.3)	23,497 (15.9)	15,749 (16.1)
East district	6,043 (2.9)	4,040 (3.0)	4,252 (2.9)	2,835 (2.9)
Income				
<22,000	50,899 (24.7)	33,986 (25.5)	37,227 (25.3)	25,592 (26.2)
≥22,000	155,159 (75.3)	99,215 (74.5)	110,223 (74.8)	72,198 (73.8)
CCI score				
0	109,100 (53.0)	64,669 (48.6)	70,596 (47.9)	45,580 (46.6)
1	56,367 (27.4)	39,175 (29.4)	43,841 (29.7)	29,177 (29.8)
2	23,682 (11.5)	16,994 (12.8)	18,982 (12.9)	13,175 (13.5)
≥3	16,909 (8.2)	12,363 (9.3)	14,031 (9.5)	9,858 (10.1)
Comorbidity^a^				
Myocardial infarction	2,236 (1.1)	1,526 (1.2)	1,965 (1.3)	1,126 (1.2)
Congestive heart failure	10,788 (5.2)	8,097 (6.1)	8,846 (6.0)	6,268 (6.4)
Peripheral vascular disease	4,049 (2.0)	2,842 (2.1)	3,197 (2.2)	2,211 (2.3)
Cerebrovascular disease	26,309 (12.8)	18,104 (13.6)	21,489 (14.6)	16,058 (16.4)
Dementia	6,873 (3.3)	4,859 (3.7)	5,197 (3.5)	4,432 (4.5)
Chronic pulmonary disease	37,250 (18.1)	26,480 (19.9)	28,212 (19.1)	19,458 (19.9)
Rheumatic disease	3,478 (1.7)	2,584 (1.9)	2,669 (1.8)	1,954 (2.0)
Peptic ulcer disease	37,970 (18.4)	28,020 (21.0)	30,034 (20.4)	20,540 (21.0)
Mild liver disease	17,117 (8.3)	12,040 (9.0)	12,846 (8.7)	8,535 (8.7)
Diabetes without chronic complication	47,561 (23.1)	33,624 (25.2)	38,861 (26.4)	24,668 (25.2)
Diabetes with chronic complication	14,090 (6.8)	10,067 (7.6)	11,750 (8.0)	7,674 (7.9)
Hemiplegia or paraplegia	950 (0.5)	582 (0.4)	721 (0.5)	553 (0.6)
Renal disease	138,47 (6.7)	9,535 (7.2)	10,790 (7.3)	7,675 (7.9)
Any malignancy, including lymphoma and leukemia, except malignant neoplasm of skin	4,637 (2.3)	2,947 (2.2)	3,158 (2.1)	2,154 (2.2)
Moderate or severe liver disease	271 (0.1)	189 (0.1)	201 (0.1)	94 (0.1)
Metastatic solid tumor	187 (0.1)	121 (0.1)	122 (0.1)	85 (0.1)
AIDS/HIV	26 (0.0)	14 (0.0)	15 (0.0)	11 (0.0)
Drug-drug Interaction		35,828 (26.9)	39,692 (26.9)	28,779 (29.4)
Polypharmacy^b^		42,711 (32.1)	47,917 (32.5)	35,839 (36.7)

PIM, potentially inappropriate medication; SD, standard deviation; CCI, charlson comorbidity index.

^a^ Within the 1 year before the first hospitalization.

^b^ Polypharmacy (>5 medications).

#### Drug-drug interactions (DDIs)

The DDI pairs were taken from those listed by McEvoy et al. (2017) and the National Institute of Health and Care Excellence (NICE) clinical guidelines (Chang et al., 2015). The DDI pairs in Micromedex^®^ provided information on the severity of contraindication; UptoDate^®^ provided DDI pairs in categories in X (Shariff et al., 2021). The DDI pairs were classified by human body systems based on the potential DDI-induced ADRs, including the cardiovascular system (bradycardia, QT prolongation, ventricular arrhythmias, and hypotensive), central nervous system (hypotensive, extrapyramidal symptoms [EPS], and central nervous system toxicity), blood (bleeding, hypoglycemia, hyperkalemia, hypokalemia, thrombotic events, and change in drug concentrations), gastrointestinal system (gastrointestinal lesions), and musculoskeletal system (myopathy). The patients’ DDI pairs were determined for the prehospitalization and post-discharge periods. The number of DDI pairs was also identified.

#### Covariates

Patients’ demographic (age and gender), socioeconomic (residential areas of NHI division, monthly income of NHI registration), Charlson Comorbidity Index (CCI), and type of comorbidity were retrieved at the first hospitalization. The insurance income ranking was used as a proxy for the monthly income level. A patient had a high or low monthly income if they received New Taiwan dollars (NT$) < 22,000 or NT$ ≥ 22,000 monthly, respectively (in January 2023, US$ 1 = approximately NT$ 30). Patients’ comorbidities were identified within the year preceding the first discharge to calculate the CCI score (Charlson et al., 1987) and further categorized into four groups (i.e., 0, 1, 2, and ≥3 comorbidities).

To determine polypharmacy, we selected only those drugs supplied for more than 28 days and prescribed within 30 days of the first discharge. Polypharmacy was defined as the concomitant use of 5 or more drugs (Lu et al., 2015).

### Statistical analysis

Logistic regression was used to evaluate the odds ratios (ORs) of clinical outcomes in the patients prescribed PIM after discharge, after adjusting for the covariates age at hospitalization, gender, geographic region, income, CCI score, admitted year, DDI (post-discharge), and polypharmacy. The results were reported as adjusted odds ratios (aORs) with 95% confidence intervals (CIs). Data management, computation, and analysis were performed using SAS software, version 9.4 (SAS Institute, Inc., Cary, NC, United States).

## Results

### Study population characteristics

As shown in Table 1, a total of 206,058 older patients (mean age: 72.5 years) were enrolled in this study. Of these, 50.2% were female, 33.4% lived in the Taipei district, and 75.3% had high income. In the analysis, PIM-Taiwan, Beers, and PRICUS criteria identified 133,201 (64.6%), 147,450 (71.6%), and 97,790 (47.5%), respectively, as having PIM exposure. The following percentage of patients had a CCI score of 0: 48.6% (in PIM-Taiwan), 47.9% (in Beers), and 46.9% (in PRISCUS). However, those assessed via PRISCUS criteria had a higher proportion of CCI scores of 2 and ≥3 (13.5% and 10.1%, respectively) than those assessed via PIM-Taiwan (12.8% and 9.3%, respectively) or Beers criteria (12.9% and 9.5%, respectively). In all three cohorts, more than 10% had the comorbidities of cerebrovascular disease, chronic pulmonary disease, peptic ulcer disease, and diabetes without chronic complications. In all three cohorts, more than one-fourth of the patients had DDIs. PIM-Taiwan, Beers, and PRICUS criteria identified polypharmacy in 32.1%, 32.5%, and 36.7% of the cohort, respectively.

### PIM patterns

**FIGURE 1:**
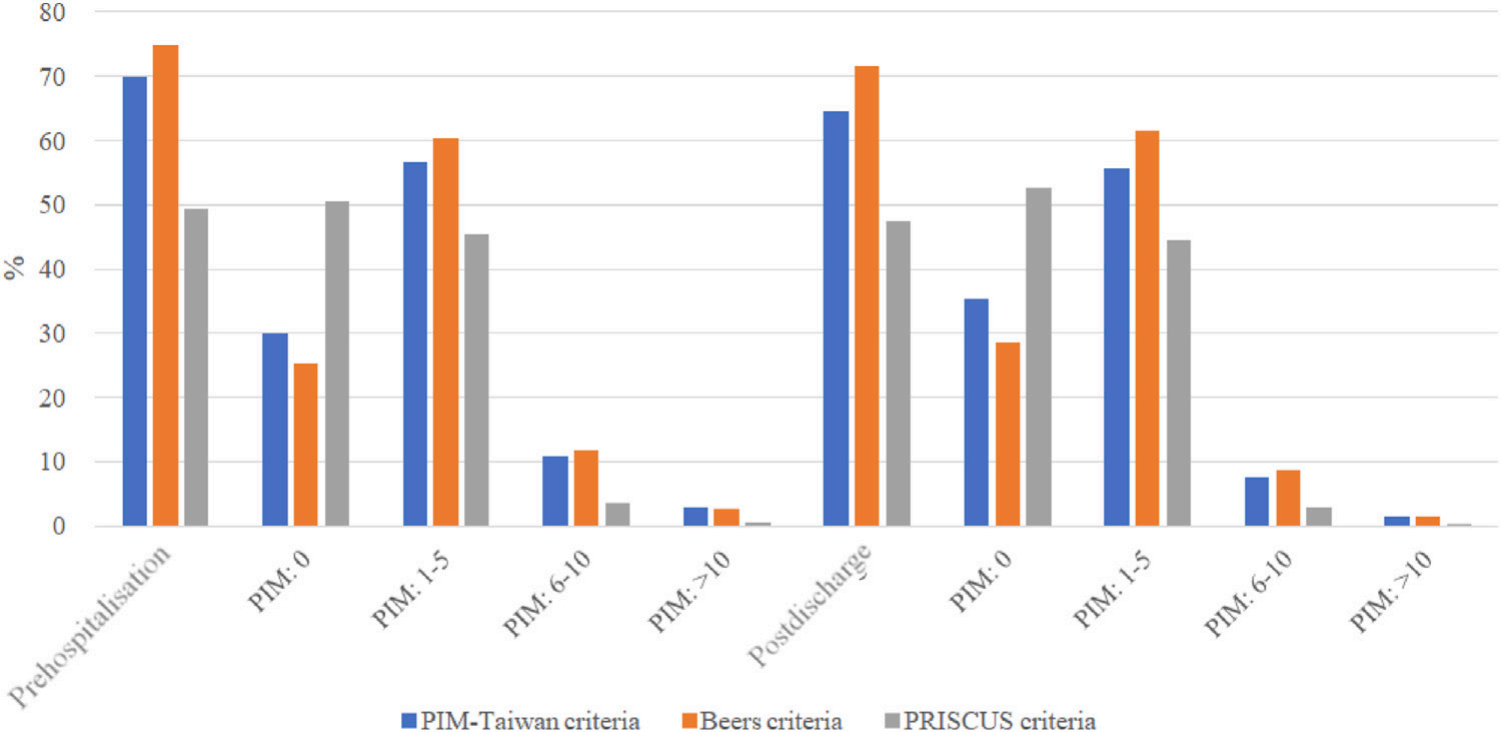
The distributions of potentially inappropriate medication at the prehospitalization and those at the postdischarge. PIM, potentially inappropriate medication; Prehospitalization, within 30 days before the first hospitalization; Postdischarge , within 30 days after the first discharge.

As shown in Figure 1, all three PIM criteria showed a lower percentage of patients with PIM exposure at post-discharge than before hospitalization. Compared to before hospitalization, fewer patients post-discharge had PIM exposure in all categories (i.e., ≥10, 6–10, or 1-5 items). As shown in Table 2, PIM-Taiwan criteria found the greatest rates of PIM exposure in all patients after their hospitalization in the central nervous (46.2%), musculoskeletal (45.6%), and gastrointestinal (23.3%) systems. Beers criteria found similar risk for PIM exposure to the musculoskeletal (63.4%), central nervous (33.6%), and gastrointestinal (21.8%) systems. In the PRISCUS criteria, the central nervous (47.0%), cardiovascular (43.2%), and musculoskeletal (27.6%) systems had the highest PIM exposure.

**TABLE 2:** Potentially inappropriate medication (PIM) exposure between all patients at postdischarge and those with 90-day rehospitalization at postdischarge.

	Postdischarge^a^	90-day rehospitalization^b^
	n (%)	n (%)
PIM-Taiwan criteria	n = 133,201	
Cardiovascular system	24,449 (18.4)	6,909 (28.7)
Endocrine system	8,645 (6.5)	2,009 (23.2)
Gastrointestinal system	31,069 (23.3)	9,033 (29.1)
Genitourinary system	3,683 (2.8)	832 (22.6)
Musculoskeletal system	60,707 (45.6)	13,645 (22.5)
Central nervous system	61,543 (46.2)	15,947 (25.9)
Respiratory system	22,261 (16.7)	6,066 (27.3)
Sex hormones	2,489 (1.9)	442 (17.8)
Beers criteria	n = 147,450	
Cardiovascular system	26,947 (18.3)	7,543 (28.0)
Endocrine system	17,760 (12.0)	4,653 (26.2)
Gastrointestinal system	32,072 (21.8)	9,863 (30.8)
Musculoskeletal system	93,538 (63.4)	21,886 (23.4)
Central nervous system	49,488 (33.6)	13,148 (26.6)
Respiratory system	15,913 (10.8)	4,135 (26.0)
Sex hormones	3,392 (2.3)	760 (22.4)
Anti-infective	174 (0.1)	42 (24.1)
Blood and blood forming organs	11,927 (8.1)	3,005 (25.2)
PRISCUS criteria	n = 97,790	
Cardiovascular system	42,199 (43.2)	11,323 (26.8)
Genitourinary system	4,243 (4.3)	972 (22.9)
Musculoskeletal system	26,974 (27.6)	5,897 (21.9)
Central nervous system	45,953 (47.0)	12,058 (26.2)
Respiratory system	8,153 (8.3)	2,676 (32.8)
Anti-infective	174 (0.2)	42 (24.1)
Blood and blood forming organs	2,537 (2.6)	677 (26.7)

^a^ Within 30 days after the first discharge.

^b^ The study outcome of 90-day rehospitalization was the detection of the following events within 90 days after the first discharge: rehospitalization, emergency department visit, hospitalization via the emergency department, and death.

### Risk of 90-day rehospitalization

The older patients with the greatest risk of 90-day rehospitalization were those exposed to PIM affecting the gastrointestinal (29.1%), cardiovascular (28.7%), and respiratory (27.3%) systems, according to PIM-Taiwan criteria; for Beers criteria, it was the gastrointestinal (30.8%), cardiovascular (28.0%), and central nervous (26.6%) systems; PRISCUS criteria found the greatest risk of 90-day rehospitalization in those with exposure to PIM affecting the respiratory (32.8%) and cardiovascular (26.8%) system, and the blood and blood-forming organs (26.7%) (Table 2).

Table 3 provides the risk of 90-day rehospitalization in older patients according to each PIM criteria. According to PIM-Taiwan criteria, exposure to PIM affecting the cardiovascular (aOR 1.37, 95% CI = 1.32–1.41), gastrointestinal (aOR 1.26, 95% CI = 1.23–1.30), central nervous (aOR 1.11, 95% CI = 1.08–1.14), and respiratory (aOR 1.16, 95% CI = 1.12–1.20) systems was significantly associated with increased risk of having 90-day rehospitalization, after adjustment for covariates; conversely, exposure to PIM affecting the sex hormones (aOR 0.74, 95% CI = 0.66–0.82), genitourinary (aOR 0.82, 95% CI = 0.76–0.89), endocrine (aOR 0.91, 95% CI = 0.86–0.96), and musculoskeletal (aOR 0.96, 95% CI = 0.93–0.98) systems was significantly associated with decreased risk. In Beers criteria, exposure to PIM affecting the cardiovascular (aOR 1.37, 95% CI = 1.32–1.41), gastrointestinal (aOR 1.38, 95% CI = 1.35–1.42), central nervous (aOR 1.18, 95% CI = 1.15–1.21), endocrine (aOR 1.10, 95% CI = 1.06–1.15), and respiratory (aOR 1.09, 95% CI = 1.04–1.13) systems was significantly positively associated with the risk of readmission, after adjustment for covariates. In PRISCUS criteria, exposure to PIM affecting the respiratory (aOR 1.48, 95% CI = 1.41–1.56), central nervous (aOR 1.12, 95% CI = 1.09–1.15), and cardiovascular (aOR 1.20, 95% CI = 1.16–1.24) systems was significantly positively associated with 90-day readmission, after adjustment for covariates; conversely, exposure to PIM affecting the genitourinary (aOR 0.82, 95% CI = 0.76–0.88) and musculoskeletal (aOR 0.86, 95% CI = 0.83–0.89) systems was significantly negatively associated with readmission.

**TABLE 3:** Risk of 90-day rehospitalization^a^ in older patients due to the systems of three PIM criteria after the first discharge.

	Yes^b^	No^c^	Crude OR	*p*-value	Adjusted OR^d^	*p*-value
	n (%)	n (%)				
PIM-Taiwan criteria						
Cardiovascular system	6,909 (28.3)	26,026 (23.9)	1.25 (1.21–1.29)	<0.0001	1.37 (1.32–1.41)	<0.0001
Endocrine system	2,009 (23.2)	30,926 (24.8)	0.92 (0.87–0.97)	0.0009	0.91 (0.86–0.96)	0.0004
Gastrointestinal system	9,033 (29.1)	23,902 (23.4)	1.34 (1.30–1.38)	<0.0001	1.26 (1.23–1.30)	<0.0001
Genitourinary system	832 (22.6)	32,103 (24.8)	0.89 (0.82–0.96)	0.0023	0.82 (0.76–0.89)	<0.0001
Musculoskeletal system	13,645 (22.5)	19,290 (26.6)	0.80 (0.78–0.82)	<0.0001	0.96 (0.93–0.98)	0.0006
Central nervous system	15,947 (25.9)	16,988 (23.7)	1.13 (1.10–1.15)	<0.0001	1.11 (1.08–1.14)	<0.0001
Respiratory system	6,066 (27.3)	26,869 (24.2)	1.17 (1.13–1.21)	<0.0001	1.16 (1.12–1.20)	<0.0001
Sex hormones	442 (17.8)	32,493 (24.9)	0.65 (0.59–0.72)	<0.0001	0.74 (0.66–0.82)	<0.0001
Beers criteria						
Cardiovascular system	7,543 (28.0)	28,608 (23.7)	1.25 (1.21–1.29)	<0.0001	1.37 (1.32–1.41)	<0.0001
Endocrine system	4,653 (26.2)	31,498 (24.3)	1.11 (1.07–1.15)	<0.0001	1.10 (1.06–1.15)	<0.0001
Gastrointestinal system	9,863 (30.8)	26,288 (22.8)	1.51 (1.47–1.55)	<0.0001	1.38 (1.35–1.42)	<0.0001
Musculoskeletal system	21,886 (23.4)	14,265 (26.5)	0.85 (0.83–0.87)	<0.0001	1.00 (0.97–1.02)	0.6894
Central nervous system	13,148 (26.6)	23,003 (23.5)	1.18 (1.15–1.21)	<0.0001	1.18 (1.15–1.21)	<0.0001
Respiratory system	4,135 (26.0)	32,016 (24.3)	1.09 (1.05–1.13)	<0.0001	1.09 (1.04–1.13)	<0.0001
Sex hormones	760 (22.4)	35,391 (24.6)	0.89 (0.82–0.96)	0.0038	0.93 (0.85–1.01)	0.0673
Anti-infective	42 (24.1)	36,109 (24.5)	0.98 (0.69–1.39)	0.9078	1.06 (0.74–1.51)	0.7533
Blood and blood forming organs	3,005 (25.2)	33,146 (24.5)	1.04 (1.00–1.09)	0.0704	1.01 (0.97–1.06)	0.557
PRISCUS criteria						
Cardiovascular system	11,323 (26.8)	13,221 (23.8)	1.18 (1.14–1.21)	<0.0001	1.20 (1.16–1.24)	<0.0001
Genitourinary system	972 (22.9)	23,572 (25.2)	0.88 (0.82–0.95)	0.0008	0.82 (0.76–0.88)	<0.0001
Musculoskeletal system	5,897 (21.9)	18,647 (26.3)	0.78 (0.76–0.81)	<0.0001	0.86 (0.83–0.89)	<0.0001
Central nervous system	12,058 (26.2)	12,486 (24.1)	1.12 (1.09–1.15)	<0.0001	1.12 (1.09–1.15)	<0.0001
Respiratory system	2,676 (32.8)	21,868 (24.4)	1.51 (1.44–1.59)	<0.0001	1.48 (1.41–1.56)	<0.0001
Anti-infective	42 (24.1)	24,502 (25.1)	0.95 (0.67–1.35)	0.7727	1.00 (0.70–1.43)	0.9925
Blood and blood forming organs	677 (26.7)	23,867 (25.1)	1.09 (1.00–1.19)	0.0619	1.02 (0.93–1.12)	0.7046

PIM, potentially inappropriate medication; OR, odds ratio; Postdischarge, within 30 days after the first discharge.

^a^ The study outcome of 90-day rehospitalization was the detection of the following events within 90 days after the first discharge: rehospitalization, emergency department visit, hospitalization via the emergency department, and death.

^b^ Had PIM, exposure and clinical outcome.

^c^ No PIM, exposure but had a clinical outcome.

^d^ Adjusted for age, gender, geographic region, income, Charlson Comorbidity Index score, drug-drug interaction, and polypharmacy.

### DDI patterns

Table 4 shows the patterns of DDIs among older adults at prehospitalization, all patients post-discharge, and at post-discharge in those with 90-day rehospitalization. The prehospitalized older adults had a slightly higher percentage of DDIs than those at post-discharge (21.6% vs. 21.4%). Subsequently, it was found that the rate of 90-day rehospitalization among individuals with DDIs at post-discharge was 14.2%. Notably, patients with three or more DDI pairs at post-discharge had a higher 90-day rehospitalization rate of 18.3% compared to those with one or two DDI pairs. The potentially harmful DDI pairs occurring at more than 1% post-discharge were those affecting bleeding (10.7%), hypotension (8.2%), myopathy (3.7%), bradycardia (1.7%), change in drug concentrations (1.6%), and hyperkalemia (1.5%) (Table 4). The most frequent types of potentially harmful DDI pairs in patients with 90-day rehospitalization were those affecting bleeding (n = 2,959; 13.4%), hypotension (n = 2,645; 15.7%), myopathy (n = 1,031; 13.6%), bradycardia (n = 539; 15.5%), change in drug concentrations (n = 556; 16.4%), and hyperkalemia (n = 546; 18.3%). However, patients with 90-day rehospitalization had higher rates of the potentially harmful DDI pairs of serotonin syndrome (n = 19; 48.8%), QT prolongation (n = 4; 30.8%), EPS (n = 102; 24.5%), and hypokalemia (n = 275; 20.1%) (Table 4).

**TABLE 4:** Drug-drug interaction patterns among older adults at prehospitalization, postdischarge, and those with 90-day rehospitalization^a^ after discharge.

	Prehospitalization	Postdischarge	90-day rehospitalization^a^
	All, n (%)^b^	All, n (%)^b^	Yes, n (%)
DDI	44,481 (21.6)	44,145 (21.4)	6,278 (14.2)
The number of DDI pairs			
1	26,310 (59.2)	29,231 (66.2)	3,828 (13.1)
2	10,610 (23.9)	9,926 (22.5)	1,539 (15.5)
≥3	7,561 (17.0)	4,988 (11.3)	911 (18.3)
DDI class^c^			
Cardiovascular system			
Bradycardia	3,387 (1.6)	3.481 (1.7)	539 (15.5)
QT prolongation	13 (0.0)	13 (0.0)	4 (30.8)
Ventricular arrhythmias	262 (0.1)	267 (0.1)	52 (19.5)
Hypotensive	15,504 (7.5)	16,870 (8.2)	2,645 (15.7)
Central nervous system			
Serotonin syndrome	48 (0.0)	39 (0.0)	19 (48.8)
EPS	1,052 (0.5)	416 (0.2)	102 (24.5)
Nervous system toxicity	325 (0.2)	267 (0.1)	53 (19.9)
Blood			
Bleeding	23,229 (11.3)	22,031 (10.7)	2,959 (13.4)
Hypoglycemia	245 (0.1)	372 (0.2)	66 (17.7)
Hyperkalemia	3,685 (1.8)	2,994 (1.5)	546 (18.3)
Hypokalemia	1,208 (0.6)	1,369 (0.7)	275 (20.1)
Thrombotic events	7 (0.0)	6 (0.0)	-^d^
Change drug concentrations	3,128 (1.5)	3,387 (1.6)	556 (16.4)
Gastrointestinal system			
Gastrointestinal lesions	328 (0.2)	80 (0.0)	13 (16.3)
Musculoskeletal system			
Myopathy	6,278 (3.1)	7,576 (3.7)	1,031 (13.6)

DDI, drug-drug interaction; EPS, extrapyramidal symptoms; Prehospitalization, within 30 days before the first hospitalization; Post-discharge, within 30 days after the first discharge.

^a^ The study outcome of 90-day rehospitalization was the detection of the following events within 90 days after the first discharge: re-hospitalization, emergency department visit, hospitalization via the emergency department, and death.

^b^ The denominator is the study cohort (*n* = 206,058).

^c^ The DDI, class was classified by human body system, based on the potential DDI-induced adverse drug reactions.

^d^ To protect the personal data of patients, any number less than three cannot be provided by the Health and Welfare Data Centre.

## Discussion

### Main study findings

To our knowledge, this is the first nationally representative evaluation of the association between exposure to PIM and health-related outcomes among older adults in Taiwan. We compared exposure to PIM and the risk of 90-day rehospitalization in this population using the PIM-Taiwan, PRISCUS, and Beers criteria systems. The PIM criteria identified a high percentage of older adults who had been prescribed at least one PIM at the first hospitalization; meanwhile, the greatest incidence of PIM was found by Beers, then PIM-Taiwan, and then PRISCUS criteria. The findings proved that Beers criteria are more efficient in predicting 90-day rehospitalization among older adults experiencing PIM in Taiwan than either PIM-Taiwan or PRISCUS criteria.

### Findings in the context of other studies

This retrospective cohort study, examining 206,058 older adults at the first hospitalization, found varying PIM rates from 48% to 72% using the PRICUS, PIM-Taiwan, and Beers criteria systems. Our findings are similar to those of previous studies (Chang et al., 2014; Storms et al., 2017). Approximately one-half of the older patients in our study had a CCI score of 0 at the first hospitalization; however, those assessed via PRISCUS criteria had a higher proportion of CCI scores of 2 and ≥3 than those evaluated using the other two criteria. The data showed that about one-half of our older participants felt well in physical condition; the results implied that the PRICUS criteria had greater sensitivity than the other two criteria in identifying participants with poor physical condition (Fromm et al., 2013; Chang et al., 2019). In addition, over one-third of our patients had polypharmacy (taking >5 medications) at the first hospitalization. Our finding was similar to that of the other study in Australia (36%) (Lu et al., 2015).

As was shown in Figure 1, older adults in Taiwan had fewer PIM prescribed after their first hospital discharge. According to Table 2, older adults in Taiwan after their first discharge had PIM exposure most commonly in the musculoskeletal, central nervous, gastrointestinal, and cardiovascular systems. The result of high PIM exposure in the musculoskeletal and gastrointestinal systems is not consistent with the results of other studies (Ye et al., 2016; Saboor et al., 2019). This discrepancy may be related to differences in medication prescribing habits in different regions. Furthermore, we initially assessed the association of PIM with 90-day rehospitalization among older adults in Taiwan. Table 2 shows that the older patients with 90-day rehospitalization still had PIM exposure in the musculoskeletal, central nervous, gastrointestinal, and cardiovascular systems after their hospitalization. We further evaluated the association of exposure to PIM with the risk of 90-day rehospitalization. According to the Beers criteria, exposure to PIM affecting the cardiovascular, gastrointestinal, central nervous, endocrine, and respiratory systems was positively associated with an increased risk of 90-day rehospitalization among older adults in Taiwan; hence, lack of PIM exposure was negatively associated with the risk. In PIM-Taiwan, PIM exposure affecting the cardiovascular, gastrointestinal, central nervous, and respiratory systems was positively associated with the risk; conversely, exposure to PIM affecting the sex hormones, genitourinary, endocrine, and musculoskeletal systems was negatively associated with the risk. In PRISCUS criteria, exposure to PIM affecting the respiratory, central nervous, and cardiovascular systems was positively associated with the risk of rehospitalization; conversely, exposed to PIM affecting the genitourinary and musculoskeletal systems had a negative association. Our findings tended to corroborate those of most studies. In a systematic review and meta-analysis including 63 studies, the pooled estimates for PIM and all-cause hospitalization were not statistically significant (aOR 1.11, 95% CI 0.76–1.63; adjusted Hazard Ratio 1.02, 95% CI 0.89–1.18) (Garcia et al., 2015). In brief, Beers criteria was proven more efficient in predicting 90-day rehospitalization among older adults experiencing PIM in Taiwan, compared to PIM-Taiwan and PRISCUS criteria.

Approximately 10%–30% of all hospitalizations in older adults are due to ADRs, of which almost 50% are potentially preventable (Leendertse et al., 2008; Salvi et al., 2012; Parameswaran Nair et al., 2016; Oscanoa et al., 2017). PIM criteria can help physicians and pharmacologists detect DDIs early and reduce DDI-induced ADRs, even though PIM does not seem to be an important cause of ADRs in older adults (Laroche et al., 2007). The DDI pairs have been classified according to major human body systems based on the potential for DDI-induced ADRs. Although the entire cohort of patients had a higher rate of DDIs than those with 90-day rehospitalization after the first discharge, the rate of ≥3 DDI pairs occurring in those with 90-day rehospitalization obviously increased from 11.3% in the whole cohort to 18.3% in those rehospitalized. This result showed that the risk of 90-day rehospitalization in older adults may be associated with the number of DDI pairs occurring, but not the number of those who experienced a DDI. We further analyzed the DDI classes in our study; the entire cohort of patients after discharge and those who experienced 90-day rehospitalization had the same 5 most common classes of DDI: bleeding, hypotension, myopathy, bradycardia, and change in drug concentrations. A greater rate of 90-day rehospitalization was associated with the potentially harmful DDI classes of serotonin syndrome, QT prolongation, EPS, and hypokalemia, as shown in Table 4. Notably, physicians and pharmacists should be well aware of the association of the above drug-related adverse effects with rehospitalization in older adults, despite their low incidence.

### Study strengths and limitations

The primary strength of the current study was its use of a large population-based cohort that enabled analysis of the association between three PIM criteria and rehospitalization among older adults experiencing PIM. The findings may help prevent medication-related hospitalization in older adults, particularly in ethnic Chinese older adults. Furthermore, this study captured almost all prescribed medications reimbursed by the NHI, meaning our database contains detailed nationwide information on medications, including the use of drug combinations.

Of course, we acknowledge several possible limitations in this study. First, it is impossible to clearly demonstrate causality between a high incidence of PIMs and the outcome measure of rehospitalization based on this retrospective data. Second, patients’ adherence to medications could not be evaluated, due to the limitations of the prescription claims databases. However, medication nonadherence would most likely have resulted in a nondifferentiated exposure misclassification, leading to possible underestimation of PIM prevalence and rehospitalization. Third, the use of some drugs, such as over-the-counter medications, alternative remedies, and herbal supplements, could not be included, because these drugs are not covered by the NHI. Fourth, three different lists were used in three different cohort of older adults. This method may have some disadvantages such as case-to-case variance bias. Although those cohorts had minor variations, we considered these factors as variables to be adjusted in the regression analysis. Finally, we did not have access to other potentially confounding factors for hospitalization among older patients such as disease severity, biochemistry data, and patient habits, such as alcohol use and tobacco consumption.

## Conclusion

This study made several important findings. First, all three instruments found high PIM rates in older adults in Taiwan, but the greatest incidence of PIM was found by Beers, then PIM-Taiwan, and then PRISCUS criteria. Second, the 5 common potential DDI-induced adverse effects in older adults in Taiwan were bleeding, hypotension, myopathy, bradycardia, and change in drug concentrations. Despite their low incidence, the potential DDI-induced adverse effects of serotonin syndrome, QT prolongation, EPS, and hypokalemia were associated with high rates of rehospitalization among older adults. Finally, Beers criteria proved most efficient in predicting 90-day rehospitalization in older adults with PIM in Taiwan, compared to PIM-Taiwan and PRISCUS criteria. The current study findings permit several recommendations to be made. Physicians and pharmacists should seek to prevent and carefully manage ADR in older adults via the PIM criteria. In particular, they should look for PIM particularly in the DDI classes of serotonin syndrome, QT prolongation, EPS, and hypokalemia, which have the greatest potential for serious adverse events, despite their low incidence.

## Data Availability

The original contributions presented in the study are included in the article/Supplementary Material, data are available from the National Health Insurance Research Database (NHIRD) published by Taiwan National Health Insurance (NHI) Bureau. Due to legal restrictions imposed by the government of Taiwan in relation to the “Personal Information Protection Act,” data cannot be made publicly available. Requests for data may be sent as a formal proposal to the NHIRD (http://nhird.nhri.org.tw).
