# Supplementary material for: Using PIM-Taiwan, PRISCUS, and Beers criteria to assess potentially inappropriate medication use among older adults with 90-day rehospitalization: a population-based study in Taiwan
Source: Front Pharmacol. 2023 Jul 13;14:1194537. doi: 10.3389/fphar.2023.1194537 (PMC10374845; doi:10.3389/fphar.2023.1194537)
Supplement: Supplementary file 1 [file Table1.pdf]

Table S1 | Drugs with ATC code in PIM-Taiwan 2018

Category	Subtypes	Drugs name (ATC code)
Cardiovascular System	Aldosterone antagonist	Spironolactone (C03DA01)
	Antiarrhythmic	Amiodarone (C01BD01)
	Central alpha blockers	Clonidine (C02AC01)
	Digitalis glycosides	Digoxin >0.125mg/day (C01AA05)
	Peripheral alpha-1 blockers	Prazosin (C02CA01), Doxazosin (C02CA04), Terazosin (G04CA03)
Endocrine system	Sulfonylureas, long-duration	Glyburide (A10BB01), Chlorpropamide (A10BB02)
	Thiazolidibediones	Pioglitazone (A10BG03)
Gastrointestinal system	H2-recepto antagonist	Cimetidine (A02BA01), Ranitidine (A02BA02), Famotidine (A02BA03), Nizatidine (A02BA04)
	Propulsives	Metoclopramide (A03FA01)
	Anticholinergic agents	Clidinium and chlordiazepoxide (A03CA02)
Genitourinary system	Drugs for urinary frequency and incontinence	Oxybutynin (G04BD04), Tolterodine (G04BD07)

Musculoskeletal system	Non-COX-2 selective nonsteroidal anti-inflammatory drugs (NSAIDs)	Indomethacin (M01AB01), Sulindac (M01AB02), Tolmetin (M01AB03), Diclofenac (M01AB05), Alclofenac (M01AB06), Etodolac (M01AB08), Acemetacin (M01AB11), Ketorolac (M01AB15), Aceclofenac (M01AB16), Piroxicam (M01AC01), Tenoxicam (M01AC02), Meloxicam (M01AC06), Ibuprofen (M01AE01), Naproxen (M01AE02), Ketoprofen (M01AE03), Fenoprofen (M01AE04), Febufen (M01AE05), Flubiprofen (M01AE09), Tiaprofenic acid (M01AE11), Mefenamic acid (M01AG01), Flufenamic acid (M01AG03), Meclofenamic acid (M01AG04), Nabumetone (M01AX01), Niflumic acid (M01AX02), Benzydamine (M01AX07), Nimesulide (M01AX17)
Central nervous system	Antidepressants (including SSRIs and SNRIs)	Paroxetine (N06AB05), Melitracen and psycholeptics (N06CA02), Maprotiline (N06AA21), Imipramine (N06AA02), Clomipramine (N06AA04), Amitriptyline (N06AA09), Doxepin (N06AA12)
	Antiparkinsonian agents	Trihexyphenidyl (N04AA01), Biperiden (N04AA02)
	Antipsychotics, first-generation (conventional)	Chlorpromazine (N05AA01), Levomepromazine (N05AA02), Fluphenazine (N05AB02), Perphenazine (N05AB03), Trifluoperazine (N05AB06), Thioridazine (N05AC02), Haloperidol (N05AD01), Flupentixol (N05AF01), Chlorprothixene (N05AF03), Pimozide (N05AG02), Loxapine (N05AH01), Sulpride (N05AL01)
	Antipsychotics, second-generation (atypical)	Ziprasidone (N05AE04), Lurasidone (N05AE05), Clozapine (N05AH02), Olanzapine (N05AH03), Amisulpride (N05AL05), Risperidone (N05AX08), Zotepine (N05AX11), Aripiprazole (N05AX12), Paliperidone (N05AX13)

	Benzodiazepines	Clonazepam (N03AE01), Diazepam (N05BA01), Medazepam (N05BA03), Oxazepam (N05BA04), Lorazepam (N05BA06), Bromazepam (N05BA08), Clobazem (N05BA09), Prazepam (N05BA11), Alprazolam (N05BA12), Nordazepam (N05BA16), Fludiazepam (N05BA17), Oxazolam (N05BA91), Flurazepam (N05CD01), Nitrazepam (N05CD02), Flunitrazepam (N05CD03), Estazolam (N05CD04), Triazolam (N05CD05), Midazolam (N05CD08), Brotizolam (N05CD09)
	Non-benzodiazepines	Zopiclone (N05CF01), Zolpidem (N05CF02), Zaleplon (N05CF03), Eszopiclone (N05CF04)
Respiratory system	First-generation antihistamines	Brompheniramine combinations (R06AB01), Triprolidine combinations (R06AX07), Dexchlorpheniramine combinations (R05X), Diphenhydramine (R06AA02), Dimenhydrinate (R06AA02), Clemastine (R06AA04), Diphenylpyraline (R06AA07), Carbinoxamine (R06AA08), Doxylamine (R06AA09), Diphenhydramine combinations (R06AA52), Diphenylpyraline combinations (R06AA57), Brompheniramine (R06AB01), Dexchlorpheniramine (R06AB02), Chlorpheniramine (R06AB04), Promethazine (R06AD02), Mequitazine (R06AD07), Buclizine (R06AE01), Cyclizine (R06AE03), Chlorcyclizine (R06AE04), Meclizine (R06AE05), Oxatomide (R06AE06), Buclizine combinations (R06AE51), Meclizine combinations (R06AE55), Homochlorcyclizine (R06AE91), Cyproheptadine (R06AX02), Phenindamine (R06AX04), Triprolidine (R06AX07), Mebhydrolin (R06AX15), Ketotifen (R06AX17), Hydroxyzine (N05BB01)
Sex hormones	Androgens	Methyltestosterone (G03BA02), Testosterone (G03BA03)

Estrogens (combinations)	Estradiol (G03CA03), Estriol (G03CA04), Estrone (C03CA07), Estradiol combinations (G03CA53), Conjugated estrogens (G03CA57), Norethisterone and estrogen (G03FA01), Hydroxyprogesterone and estrogen(G03FA02), Progesterone and estrogen (G03FA04), Norgestrel and estrogen (G03FA10), Medroxyprogesterone and estrogen (G03FA12)

Table S2 | Drugs with ATC code in Beers criteria 2019

Category	Subtypes	Drugs name (ATC code)
Cardiovascular System	Peripheral alpha-1 blockers	Doxazosin(C02CA04), Prazosin(C02CA01), Terazosin(G04CA03)
	Central alpha-agonists	Clonidine(C02AC01), Methyldopa (C02AB01), **Guanabenz* (N/A), Guanfacine* (C02AC02)**, Reserpine >0.1mg/day(C02AA02)
	Cardiovascular	Disopyramide (C01BA03), Dronedarone (C01BD07), Digoxin (C01AA05), Nifedipine immediate release (C08CA05), Amiodarone (C01BD01)
Endocrine system	Insulins	Human insulin (A10AB01, A10AC01), Pork insulin (A10AB03, A10AC03, A10AC30), Insulin lispro (A10AB04), Insulin aspart (A10AB05), Fast-acting insulin combinations (A10AB30)
	Sulfonylureas	Chlorpropamide (A10BB02), Glimepiride (A10BB12), Glyburide (A10BB01)
Gastrointestinal system	Gastrointestinal	Metoclopramide (A03FA01), **Mineral oil oral* (A06AA01)**
	Proton-pump inhibitors (PPIs)	Omeprazole (A02BC01), Pantoprazole (A02BC02), Lansoprazole (A02BC03), Rabeprazole (A02BC04), Esomeprazole (A02BC05), Dexlansoprazole (A02BC06)
	Antispasmodics	Atropine excludes ophthalmic (A03BA01)
	Belladonna alkaloids	Butylscopolamine (A03BB01), Methylatropine (A03BB02), Belladonna with psycholeptics (A03CB), belladonna total alkaloids and psycholeptics (A03CB02), Hyoscyamine and psycholeptics (A03CB31), Butylscopolamine and analgesics (A03DB04), Clidinium-chlordiazepoxide (A03CA02), Dicyclomine (A03AA07), Homatropine excludes ophthalmic (A03BA91), Hyoscyamine (A03BA03), Methylscopolamine (A03BB03), Propantheline (A03AB05), Scopolamine (A04AD01)

Musculoskeletal system	Non-steroidal anti-inflammatory drugs (NSAIDs)	Aspirin >325mg/day (B01AC06), Diclofenac (M01AB05), Diflunisal (N02BA11), Etodolac (M01AB08), Fenoprofen (M01AE04), Ibuprofen (M01AE01), Ketoprofen (M01AE03), Meclofenamate (M01AG04), Mefenamic acid (M01AG01), Meloxicam (M01AC06), Nabumetone (M01AX01), Naproxen (M01AE02), **Oxaprozin* (M01AE12)**, Piroxicam (M01AC01), Sulindac (M01AB02), Tolmetin (M01AB03), Indomethacin (M01AB01), Ketorolac includes parenteral (M01AB15)
	Skeletal muscle relaxants	Carisoprodol (M03BA02), Chlorzoxazone (M03BB03), Cyclobenzaprine (M03BX08), **Metaxalone* (N/A)**, Methocarbamol (M03BA03), Orphenadrine (M03BC01)
	Others	Meperidine=Pethidine (N02AB02)
Central nervous system	Antidepressants	Amitriptyline (N06AA09), **Amoxapine*** (N06AA17), Clomipramine (N06AA04), **Desipramine*** (N06AA01), Doxepin >6mg/day (N06AA12), Imipramine (N06AA02), **Nortriptyline*** (N06AA10), Paroxetine (N06AB05), Protriptyline* (N06AA11), **Trimipramine*** (N06AA06)
	Antipsychotics, first-generation (conventional)	Chlorpromazine (N05AA01), Levomepromazine (N05AA02), Fluphenazine (N05AB02), Perphenazine (N05AB03), Trifluoperazine (N05AB06), Thioridazine (N05AC02), Haloperidol (N05AD01), Flupentixol (N05AF01), Chlorprothixene (N05AF03), Pimozide (N05AG02), Loxapine (N05AH01), Sulpride (N05AL01)
	Antipsychotics, second-generation (atypical)	Ziprasidone (N05AE04), Lurasidone (N05AE05), Clozapine (N05AH02), Olanzapine (N05AH03), Amisulpride (N05AL05), Risperidone (N05AX08), Zotepine (N05AX11), Aripiprazole (N05AX12), Paliperidone (N05AX13)

	Barbiturates	Amobarbital(N05CA02), **Butabarbital* (N/A), Butalbital* (N/A), Methylphenobarbital* (N03AA01),** Pentobarbital (N05CA01), Phenobarbital (N03AA02), Secobarbital (N05CA06)
	Benzodiazepines	Alprazolam (N05BA12), Estazolam (N05CD04), Lorazepam (N05BA06), Oxazepam (N05BA04), Temazepam (N05CD07), Triazolam (N05CD05), Chlordiazepoxide (N05BA02), Chlordiazepoxide in combination with Amitriptyline or Clidinium (N06CA01), Clonazepam (N03AE01), Clorazepate (N05BA05), Diazepam (N05BA01), Flurazepam (N05CD01), **Quazepam* (N05CD10)**
	Non-benzodiazepines	Eszopiclone(N05CF04), Zaleplon(N05CF03), Zolpidem(N05CF02)
	Antiparkinsonian	Benztropine oral (N04AC01), Trihexyphenidyl (N04AA01)
	Ergot alkaloids	Ergoloid mesylates (C04AE01)
	Others	Isoxsuprine (C04AA01), Meprobamate (N05BC01)
Respiratory system	First-generation antihistamines	Brompheniramine (R06AB01), Carbinoxamine (R06AA08), Chlorpheniramine (R06AB04), Chlorphenamine combination (R06AB54), Clemastine (R06AA04), Cyproheptadine (R06AX02), **Dexbrompheniramine* (R06AB06)**, Dexchlorpheniramine (R06AB02), Dimenhydrinate (R06AA02), Diphenhydramine oral (R06AA02), Doxylamine (R06AA09), Hydroxyzine (N05BB01), Meclizine (R06AE05), Promethazine (R06AD02), **Pyrilamine* (R06AC01)**, Triprolidine(R06AX07)
Sex hormones	Androgens	Methyltestosterone (G03BA02), Testosterone (G03BA03)

	Estrogens	Estradiol (G03CA03), Estriol (G03CA04), estradiol combinations (G03CA53), Conjugated estrogens (G03CA57), Norethisterone and estrogen (G03FA01), Hydroxyprogesterine and estrogen (G03FA02), Ethisterone and estrogen (G03FA03), Progesterone and estrogen (G03FA04), Norgestrel and estrogen (G03FA10), Medroxyprogesterone and estrogen (G03FA12)
	Progesterone	Megestrol (L02AB01)
	Other hormones	Somatropin (H01AC01), Thyroid (H03AA05)
Anti-infective	Anti-infective	Nitrofurantoin (J01XE01)
Blood and blood-forming organs	Antithrombotics	Dipyridamole oral short-acting does not apply to the extended-release combination with aspirin (B01AC07)
Genitourinary system	Genitourinary	Desmopressin (H01BA02)

*Drug names with bold font and star marks are unavailable in Taiwan. N/A=ATC code not available.

Table S3 | Drugs with ATC code in PRISCUS criteria

Category	Subtypes	Drugs name (ATC code)
Cardiovascular System	Antihypertensive agents and other cardiovascular drugs	Clonidine (C02AC01), Doxazosin (C02CA04), Methyldopa (C02AB01), Methyldopa (C02AB02), Nifedipine (C08CA05), Prazosin (C02CA01), Reserpine (C02AA02), Terazosin (G04CA03)
	Antiarrhythmic	Digoxin (C01AA05), Flecainide (C01BC04), Metildigoxin (C01AA08), Quinidine (C01BA01), Sotalol (C07AA07)
Genitourinary system	Anticholinergic drugs, Genitourinary	Oxybutynin (G04BD04), Solifenacin (G04BD08), Tolterodine (G04BD07)
Musculoskeletal system	Muscle relaxants	Baclofen (M03BX01)
	NSAID	Acemetacin (M01AB11), Etoricoxib (M01AH05), Indometacin (M01AB01), ketoprofen (M01AE03), Meloxicam (M01AC06), Pethidine (N02AB02), Phenylbutazone (M01AA01), Piroxicam (M01AC01)
Central nervous system	Antidepressants	Amitriptyline (N06AA09), Clomipramine (N06AA04), Doxepin (N06AA12), Fluoxetine (N06AB03), Imipramine (N06AA02), Maprotiline (N06AA21)
	Anti-dementia drugs	Nicergoline (C04AE02), Pentoxifylline (C04AD03), Piracetam (N06BX03)
	Antiepileptic drugs	Phenobarbital (N03AA02)
	Antipsychotics	Clozapine (N05AH02), Fluphenazine (N05AB02), Haloperidol (N05AD01), Levomepromazine (N05AA02), Olanzapine (N05AH03), Perphenazine (N05AB03), Thioridazine (N05AC02)
	Ergotamine	Dihydroergotoxin (C04AE01), Ergotamine (N02CA52), Ergotamine (N02CA72)

	Sedatives, hypnotic agents	Alprazolam (N05BA12), Bromazepam (N05BA08), Brotizolam (>0.125 mg/d) (N05CD09), Chlordiazepoxide (N05BA02), Clobazam (N05BA09), Diazepam (N05BA01), Diphenhydramine (R06AA02), Dipotassium clorazepate (N05BA05), Doxylamine (R06AA09), Flunitrazepam (N05CD03), Flurazepam (N05CD01), Lorazepam (>2 mg/d) (N05BA06), Lormetazepam (>0.5 mg/d) (N05CD06), Medazepam (N05BA03), Nitrazepam (N05CD02), Oxazepam (>60 mg/d) (N05BA04), Prazepam (N05BA11), Temazepam (N05CD07), Triazolam (N05CD05), Zaleplon (N05CF03), Zolpidem (>5 mg/d) (N05CF02), Zopiclone (>3.75 mg/d) (N05CF01)
Respiratory	Antihistamine	Chlorpheniramine (R06AB02), Clemastine (R06AA04), Dimetindene (R06AB03), Hydroxyzine (N05BB01), Triprolidine (R06AX07)
Anti-infective	Antibiotics	Nitrofurantoin (J01XE01)
Blood and blood-forming organs	Inhibitors of platelet aggregation	Prasugrel (B01AC22), Ticlopidine (B01AC05)
Gastrointestinal system	Antiemetic drugs	Dimenhydrinate (R06AA02)
